# Soil Does Not Explain Monodominance in a Central African Tropical Forest

**DOI:** 10.1371/journal.pone.0016996

**Published:** 2011-02-10

**Authors:** Kelvin S. -H. Peh, Bonaventure Sonké, Jon Lloyd, Carlos A. Quesada, Simon L. Lewis

**Affiliations:** 1 School of Geography, University of Leeds, Leeds, United Kingdom; 2 Conservation Science Group, Department of Zoology, University of Cambridge, Cambridge, United Kingdom; 3 Plant Systematic and Ecology Laboratory, Higher Teacher's Training College, University of Yaoundé I, Yaoundé, Cameroon; 4 School of Geography, Planning and Environmental Management, University of Queensland, St. Lucia, Queensland, Australia; Centre National de la Recherche Scientifique, France

## Abstract

**Background:**

Soil characteristics have been hypothesised as one of the possible mechanisms leading to monodominance of *Gilbertiodendron dewerei* in some areas of Central Africa where higher-diversity forest would be expected. However, the differences in soil characteristics between the *G. dewevrei*-dominated forest and its adjacent mixed forest are still poorly understood. Here we present the soil characteristics of the *G. dewevrei* forest and quantify whether soil physical and chemical properties in this monodominant forest are significantly different from the adjacent mixed forest.

**Methodology/Principal Findings:**

We sampled top soil (0–5, 5–10, 10–20, 20–30 cm) and subsoil (150–200 cm) using an augur in 6 × 1 ha areas of intact central Africa forest in SE Cameroon, three independent patches of *G. dewevrei-dominated* forest and three adjacent areas (450–800 m apart), all chosen to be topographically homogeneous. Analysis – subjected to Bonferroni correction procedure – revealed no significant differences between the monodominant and mixed forests in terms of soil texture, median particle size, bulk density, pH, carbon (C) content, nitrogen (N) content, C:N ratio, C:total NaOH-extractable P ratio and concentrations of labile phosphorous (P), inorganic NaOH-extractable P, total NaOH-extractable P, aluminium, barium, calcium, copper, iron, magnesium, manganese, molybdenum, nickel, potassium, selenium, silicon, sodium and zinc. Prior to Bonferroni correction procedure, there was a significant lower level of silicon concentration found in the monodominant than mixed forest deep soil; and a significant lower level of nickel concentration in the monodominant than mixed forest top soil. Nevertheless, these were likely to be the results of multiple tests of significance.

**Conclusions/Significance:**

Our results do not provide clear evidence of soil mediation for the location of monodominant forests in relation to adjacent mixed forests. It is also likely that *G. dewevrei* does not influence soil chemistry in the monodominant forests.

## Introduction

Some areas of tropical lowland forests are dominated by a single tree species despite tropical forests often being perceived as systems with highly diverse and complex communities [1]. In Central Africa, such low-diversity forests are often dominated by *Gilbertiodendron dewevrei*, a highly shade tolerant species that occurs across central Africa [2]. These monodominant forests exist alongside higher-diversity forests often with sharp boundaries. Studies have shown that the monodominant *G. dewevrei* forests and their adjacent mixed forests do not differ significantly in their above-ground biomass [3], wood production [4], number of stems [5] and species richness of tree ≥10 cm diameter at breast height [5].

One obvious hypothesis relating to the dominance of *G. dewevrei* is that it is a specialist on a particular soil type. The importance of edaphic conditions in the spatial distributions of tropical tree species has been well studied [6]. In contrast, there are only few studies looking at the role of soil nutrients in the spatial distribution of the monodominant forests where higher diversity would be expected [7], [8]. For example, Torti et al. (2001) showed that the soils beneath the monodominant *Gilbertiodendron* forest had lower availability of nitrogen when compared to the adjacent mixed forest [9]. More specific, the nutrient supply rate of ammonium and nitrate in the soils of the monodominant forest was lower than those of the mixed forest.

Contradictory to the findings of Torti et al. (2001), Hart (1985) and Conway (1992), however, showed that there were no significant differences in the studied soil parameters (including nitrogen) between the *G. dewevrei* forest and the adjacent high-diversity forest in the same study area – Ituri – as Torti et al. (2001) [2], [10]. In the studies of other monodominant forests, Nascimento & Proctor (1997) found no evidence that the soil determines the boundaries between the *Peltogyne gracilipes*-dominated forest and the adjacent high-diversity forest on Maraca Island, Brazil [11]. Martijena (1998) found that there were no significant differences in soil properties between the monodominant forest of *Celaenodendron mexicanum* and the adjacent high-diversity forest in Mexico [12]. Similarly, Henkel (2003) had the same findings (i.e. no edaphic difference) for the *Dicymbe corymbosa* system in Guyana [13].

Instead of the soils as the determinant of vegetation types in a landscape, Tilman (1982) predicts that tree species may alter the composition of soil nutrients when the nutrients are limiting [14] (resource-ratio hypothesis; but see Powers et al. 2004 [15]). Indeed, some studies have demonstrated that vegetation can modify the soil environments and thus drive the difference in soils in temperate forests [16] or agroforestry plantations in the tropics [17]. In a study investigating the phenomenon of monodominance, Torti et al. (2001) proposed that in the *G. dewevrei* forests, having low nutrient turnover is one of the prerequisites to achieve monodominance [9]. One way to achieve the slowing of nutrient turnover is by producing poor-quality leaf litter that is slow to decompose. The resulting slow rate of nutrient turnover might lower the nutrient availability to plants and in turn affect the survival of some species in the *G. dewevrei* forests. Torti *et al*. (2001) found that the leaf litters of *G. dewevrei* in the *G. dewevrei* forests tended to accumulate to a mass of three times more than that of the high-diversity forests at Ituri, [9]. Concomitant with litter accumulation is a lower rate of decomposition in the *G. dewevrei* forests in which leaf litter decomposed two to three times slower in the monodominant forests than in the high-diversity forests [9]. Given the large amount of litterfall accumulated on the ground of the *G. dewevrei* forests, and their slow decomposition rates of the leaf litters, it follows that these litters may release a lower concentration of nutrients into the soils, perhaps changing the soil properties of these forests.

In this study, we investigated the physical and chemical soil properties from the *G. dewevrei* forests (hereafter called the monodominant forests) and the adjacent high-diversity forests (hereafter called the mixed forests) contiguous with the main block of the Congo Basin forest block, in Dja Faunal Reserve, South East Cameroon, to test whether there are differences in soil properties between the two forest types. This study investigated if the micronutrient levels are different between these two forest types in Africa. We suggest that the association between soil properties and the occurrence of the *G. dewevrei* forests remain equivocal and worth further investigation in our study area of the same forest type dominated by *G. dewevrei* approximately 1000 km from Ituri. Potential differences in soil properties between the two forest types may explain the distributions of *G. dewevrei* within Central African forest, and potentially explain some differences in ecosystem functioning such as net primary productivity differences between the two forest types. Here we investigate if the availability of nutrients for *G. dewevrei* uptake was different in comparison to the adjacent high-diversity forests.

## Materials and Methods

### Study Area

**Figure 1:**
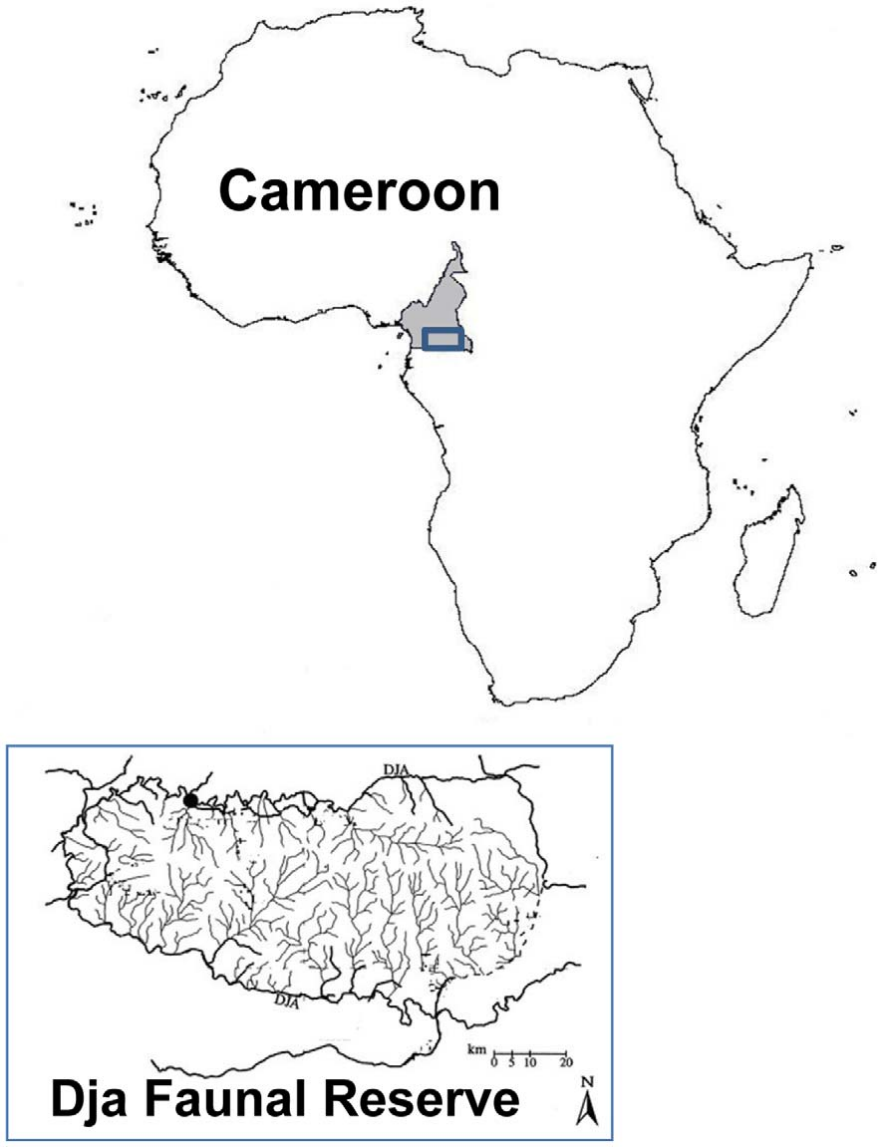
Map of study location at Dja Faunal Reserve in Cameroon.

Our study was conducted at the Dja Faunal Reserve (hereafter called Dja), located between 2°49′–3°23′N and 12°25′–13°35′E in south-eastern Cameroon (Fig. 1). The reserve was established in 1950 and is one of the largest protected rain forest areas in Africa [18]. The reserve covers an area of 526000 ha, which consists of lowland moist evergreen tropical forests at an elevation between 400–800 m [18]. About two-third of the reserve's perimeter is demarcated by the Dja River, forming a natural boundary. Only the south-east part of the reserve is not being encircled by the Dja River. Such inaccessibility due to the natural barrier offers the reserve protection from large-scale human disturbance. The Dja River flows in an anti-clockwise direction around the reserve and eventually empties into the Sangha River in the Republic of Congo [19]. Within the reserve, there is a complex hydrological network (Fig. 1). The nearby towns are Lomié (situated at 5 km to the east of the reserve), Bengbis (about 10 km to the northwest) and Messamena (about 45 km to the north) [19].

The climate of the reserve is of equatorial. Based on the meteorological data collected from three locations near the reserve (Akonolinga [2°56′N, 11°57′E], Sangmélima [3°47′N, 12°15′E] and Lomié [3°09′N, 13°37′E]) between 1979 and 2008, the multiannual mean annual rainfall is 1441 mm, 1575 mm and 1520 mm (average value  = 1512 mm), respectively. The average monthly rainfall ranges from 18 mm in December and January to 268 mm in October. The climate is characterized by two wet seasons with rainfall peaks in May (average monthly rainfall: 191 mm) and October (average monthly rainfall: 268 mm). The two dry periods are July–August (113–122 mm) and December–February (18–27 mm). The maximum average monthly temperature in the reserve is 25.8°C in February, and minimum average monthly temperature is 23.6°C in October.

Crystalline metamorphic rocks, comprising schists, gneisses and quartzite, from Precambrian origin form the underlying substratum of the reserve [18]. Soils of the region are often described as clayey and poor in nutrients [18]. Although a recent study by van Gemerden (2003) suggests that lowland rain forests in southern Cameroon may have experienced historical anthropogenic disturbances [20], the reserve has no evidence of major recent human-induced disturbance (e.g., logging, clearance). The vegetation in the reserve has a main canopy of 30–40 m with tree emergents rising to 60 m [18]. Sonké (2004) recorded at least 372 tree species with diameter at breast height (dbh) ≥10 cm [21]. The predominance of Euphorbiaceae (18% of all recorded species) in the reserve is a characteristic commonly shared by African tropical forests elsewhere [21]. There are about 58 species that form the basic flora composition of the canopy in the reserve [18]. Large naturally-occurring monodominant patches of *G. dewevrei* occur within the mixed forest throughout the reserve. Although the size of these monodominant forest patches in the reserve is not known, *G. dewevrei* often extensively dominates on the plateau of central Africa [9]. The common tree species in the mixed forests include: *Anonidium mannii*, *Carapa procera*, *Petersianthus macrocarpus*, *Polyalthia suaveolens* and *Tabernaemontana crassa* [21]. Besides the monodominant *G. dewevrei* forest and the mixed forest, there is also swamp vegetation characterized mainly by genera *Eremospatha*, *Laccosperma*, *Oncocalamus* and *Raphia* [21].

The reserve harbours important populations of mammals, including elephants and lowland gorillas [22], and birds [23]. At least 78 species of mammals and 320 species of birds are recorded in the reserve [19]. Communities of nomadic hunter-gatherer Baka indigenous people inhabit the reserve, alongside a small number of sedentary Badjoué, Bantou, Boulou, Fang and Nzimé people who engage in subsistence agriculture near the edges of the reserve [19].

### Soil Sampling

Soil was collected from three monodominant forests and their adjacent mixed forests within 100 m ×100 m (1 ha) plot surveys. All three plots in *Gilbertiodendron* forest were located in independent *Gilbertiodendron* patches identified using satellite images. The locations of the *Gilbertiodendron* patches were at least 4 km apart from each other. For each monodominant forest plot, a corresponding plot was also established in the adjacent mixed-species forest for comparative purposes. The three mixed forest plots were 452 m, 505 m and 818 m away from their *Gilbertiodendron*-dominated counterparts. In total, six 1 ha plots were demarcated.

We sampled soil at three locations within each of the six plots. We sampled soils at two stratified-random points within each plot (based on topography within the plot) at five depths: at 0–5 cm, 5–10 cm, 10–20 cm, 20–30 cm, and 150–200 cm using a soil auger (Eijkelkamp Agrisearch Equipment BV, Giesbeek, The Netherlands). At a point along the perimeter of each plot, representing the median topographic conditions, we dug a soil pit of 2 m deep to collect the soil samples at the five depths. Therefore, in total, we sampled soils at five depths at three different locations within each of the six plots for the soil analyses. After sampling, the soils were placed in plastic bags, sealed, and then air-dried at room temperature, followed sieving through a 2 mm mesh ready for physical and chemical analysis.

Samples for bulk density determinations were obtained from the wall of the soil pit in each of the six sampling plots. Samples were taken using container-rings of known volume (Eijkelkamp Agrisearch Equipment BV, Giesbeek, The Netherlands). One sample from each depth (0–5, 5–10, 10–20, 20–30, 150–200), of known volume, was oven-dried at 105°C to constant dry mass. Bulk density was then determined as a measure of the dry mass per unit volume (g cm^−3^).

### Soil Laboratory Analysis

Particle size was measured on the sodium dithionite and sodium citrate treated soil. Mixture of 4 g of soil with 2 g of sodium dithionite, 22 g of sodium citrate and 100 ml of deionized water were shaken overnight and then allowed to settle for 12 hrs. We decanted the liquid and added 100 ml deionized water with 1 g of calgon. The mixture was again shaken for one hour before being introduced to the particle size analyzer (Coulter LS 230, Coulter Electronics Limited) for the determination of the median value of particle size. We followed the International Society of Soil Science size classes for the soil particle size classification: particle size <0.002 mm was classified as clay, ≥0.002 mm and <0.02 mm was classified as silt, and >0.02 mm was classified as sand [24]. We then determined the proportions of clay, silt and sand for each soil sample using gravimetry [25].

Soil pH was measured on a mixture of 10 g of soil with 25 ml of deionized water. Readings of the pH meter were taken only after the mixtures were stirred for one hour. Total nitrogen (N) and carbon (C) contents in soils were measured on the finely ground samples using elemental analyzer (Euro EA, EuroVector instruments and software). We determined the concentrations of aluminium (Al), calcium (Ca), potassium (K), magnesium (Mg) and sodium (Na) in the soil samples by using a single extraction with silver-thiourea for measuring exchangeable cations. The exchangeable cations were extracted for 4 hrs from 5 g samples by 30 ml of silver-thiourea reagent and analysed by inductively coupled plasma optical emission spectrophotometer (Optima S300 DV, Perkin Elmer) [26]. Concentrations of another 16 elements – boron(B), barium (Ba), cobalt(Co), chromium(Cr), copper(Cu), iron (Fe), manganese (Mn), molybdenum (Mo), nickel (Ni), selenium (Se), silicon (Si), strontium (Sr), titanium (Ti), vanadium (V) and zinc (Zn) – were also measured using same cation exchange capacity (CEC) procedure. Although this method was not optimal for quantitative analyses of these elements, using a standardized method enabled us to compare the relative quantities of these elements in the soils of the two forest types.

Inorganic phosphorus (P) and total P were extracted using NaOH by a fractionation method in which the former was precipitated with 0.9 M H_2_SO_4_ and the latter was treated with ammonium persulphate and H_2_SO_4_ digests on a hotplate (<400°C) [27]. Then the concentrations of inorganic NaOH-extractable P and total NaOH-extractable P were determined by the method following Murphy and Riley (1962) [28]. We also extracted the labile P using resin strips in the mixture of 0.5 g samples with 30 ml deionized water. The resin strips were then removed and soaked in 20 ml 0.5 N HCL before the determination of labile P concentration following Murphy & Riley (1962) [28].

### Statistical Analysis

Soil property values from each of the three sampling points within each plot were averaged for each of the five soil depths (but see Dataset S1for those that were averaged using less than three sampling points). To examine if there were differences in soil properties between the monodominant and mixed forests, we calculated their mean values and 95% confidence intervals (n = 3) for each soil depth [29]. To further compare the soil properties between the two forest types, we also calculated the 95% confidence intervals for the deep soil (150–200 cm) and the averaged values of the top soil depth classes (0–5 cm, 5–10 cm, 10–20 cm and 20–30 cm) for each forest types. In addition, we compared the soil characteristics between the two forest types using paired *t* test for matched pairs (n = 3) for both top and deep soils. These multiple tests of significance were subjected to Bonferroni correction procedure [30].

## Results and Discussion

Low nutrient availability has been suggested to play a role in the formation of monodominant forests [9]. Concomitant with this soil-related mechanism is a lower rate of litter decomposition in the monodominant forests [9], [31]. Furthermore, many monodominant species are associated with ectomycorrhizae which allow more efficient exploitation of larger volumes of soils or directly decompose leaf litter [13]. Our results, however, highlight the discrepancy between this low-nutrient hypothesis and the empirical observations in *G. dewevrei*-dominated forests.

**Figure 2:**
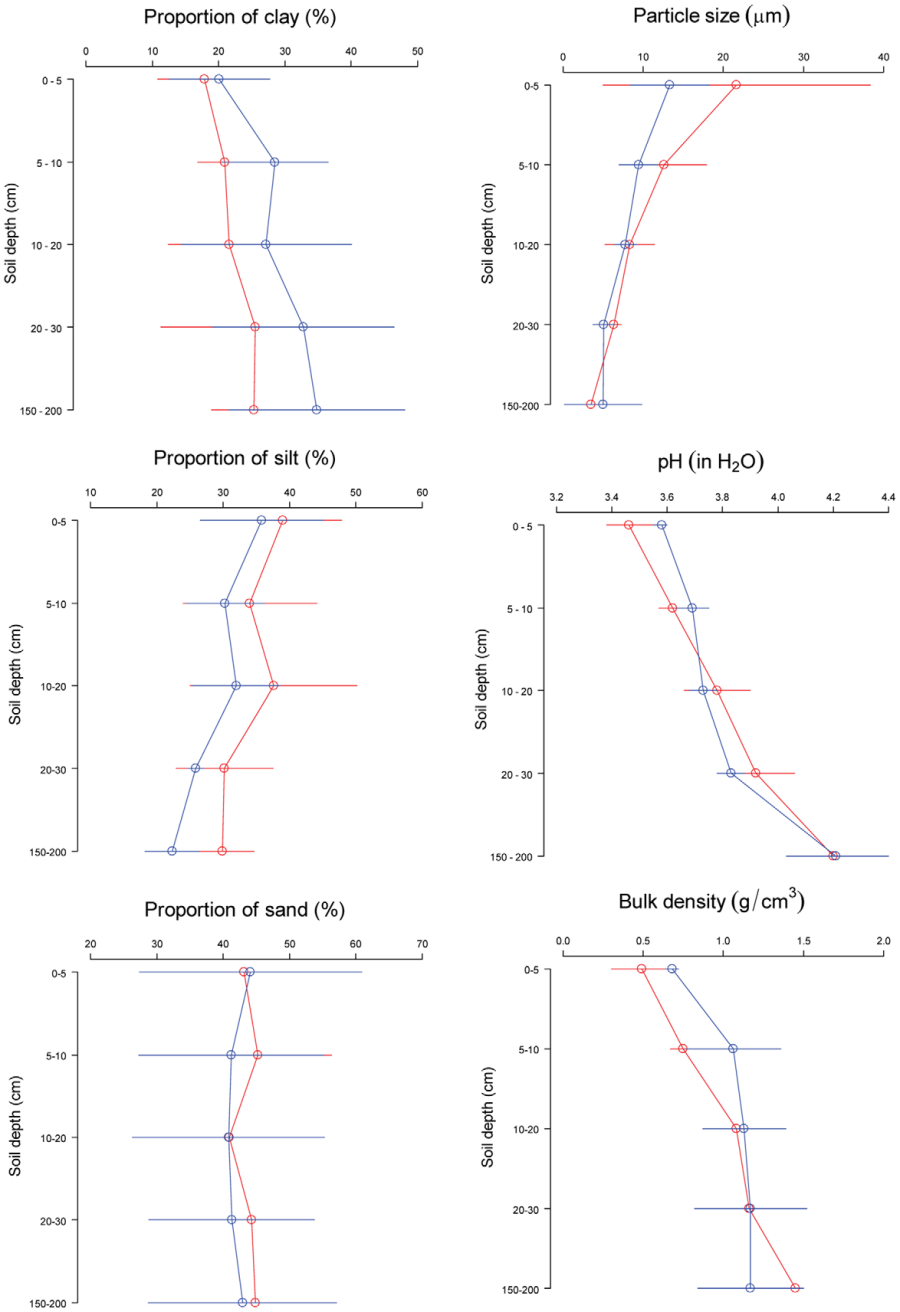
Comparison of the proportion of clay (top left); proportion of silt (middle left); proportion of sand (bottom left); particle size (top right); pH in H_2_O (middle right); and bulk density (bottom right) found in soils at different depths sampled beneath stands of forest dominated the species *Gilbertiodendron* (red line) and adjacent higher-diversity forests where no species dominates (blue lines). Circles show mean values and error bars indicate 95% confidence intervals (n = 3).

The soils from under the monodominant forests and adjacent mixed forests in the Dja Faunal reserve were both acidic, weathered clayey Ferrosols in World Reference Base for Soil Resources classification [32] (also known as Oxisols in USDA classification [33]; for detailed descriptions see Table S1). The top soil (0–30 cm) from both forest types was sandy clay loam and became sandy clay below 150 cm. The monodominant forests had consistently lower proportion of clay and higher proportion of silt than the mixed forests along the depth gradient, but the differences were not statistically significant (Fig. 2). The median soil particle size decreased with depth in both forest types (Fig. 2).

**Figure 3:**
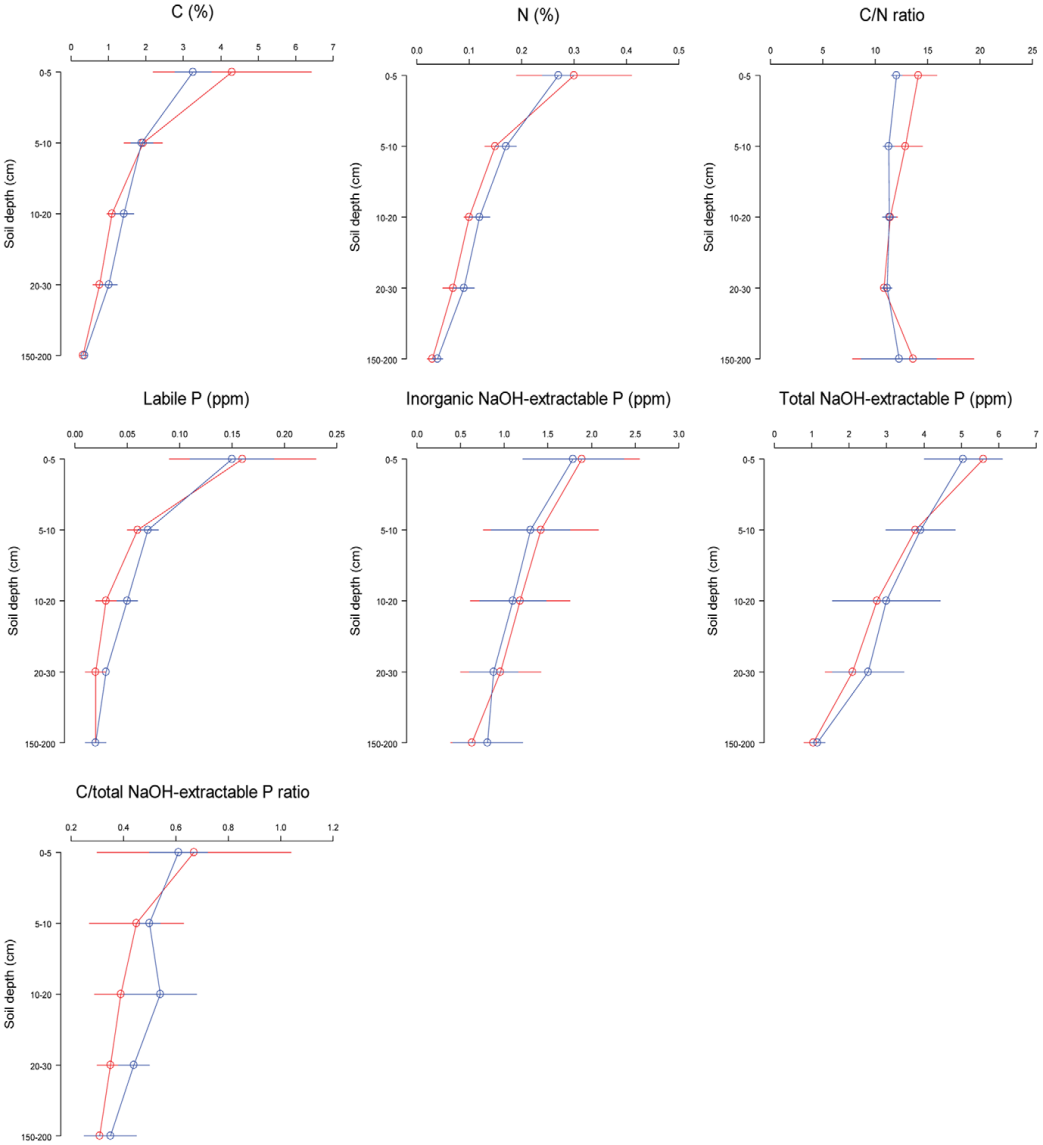
Comparison of the C content of soil organic residues (first row, left); N content of soil organic residues (first row, centre); C/N ratio (first row, right); labile P concentration (second row, left); inorganic NaOH-extractable P concentration (second row, centre); total NaOH-extractable P concentration (second row, right) and C:total NaOH-extractable P ratio (third row, left) found in soils at different depths sampled beneath stands of forest dominated the species *Gilbertiodendron* (red line) and adjacent higher-diversity forests where no species dominates (blue lines). Circles show mean values and error bars indicate 95% confidence intervals (n = 3).

Soil characteristics varied with depth. The median grain size of all soil samples from the different depths was classified as fine silt. Soil pH increased with depth in both forest types with all soil samples being acidic (Fig. 2). There were no significant differences in pH with depth from soils from under the two forest types. Similarly, higher soil bulk density was observed in greater depth in both monodominant forests and mixed forests, and not significantly different when comparing soils under the two forest types (Fig. 2). Carbon content, N content, labile P concentration, inorganic NaOH-extractable P concentration and total NaOH-extractable P concentration each declined with depth, as expected, and none were significantly different between the soils under the two forest types at any depth (Fig. 3). The C:N ratios of all soil samples from different depth ranged below 25:1 (Fig. 3; Table 1). This means that the decomposition of soil organic matter was not limited by the amount of soil N availability in both forest types. There was no significant difference between the two forest types for C:total NaOH-extractable P ratio (Fig. 3; Table 1).

**Table 1:** Soil physical and chemical characteristics in three 1 ha plots of monodominant *Gilbertiodendron* forest and three 1 ha plots of mixed forest at Dja Faunal Reserve, Cameroon.

Parameters	Top soil (0–30 cm)			Deep soil (150–200 cm)		
	Monodominant	Mixed		Monodominant	Mixed	
Proportion of clay (%)	21.5±8.1	27.2±10.4	ns	25.3±6.4	34.8±13.3	ns
Proportion of silt (%)	35.2±7.6	31.0±4.4	ns	29.9±4.8	22.3±4.1	ns
Proportion of sand (%)	43.3±7.7	41.8±14.4	ns	44.9±2.9	42.9±14.2	ns
Median particle size (µm)	12.23±6.36	8.90±2.09	ns	3.50±2.5	5.02±4.84	ns
Bulk density	0.87±0.02	1.01±0.20	ns	1.45±0.04	1.17±0.33	ns
pH (H_2_O)	3.70±0.09	3.71 ±0.04	ns	4.20±0.14	4.21±0.18	ns
C (%)	2.03±0.66	1.89±0.20	ns	0.32±0.1	037±0.07	ns
N (%)	0.15±0.03	0.16±0.01	ns	0.03±0.01	0.04±0.01	ns
C/N	12.31±1.05	11.45 ±0.45	ns	13.06±5.78	12.26 ±3.60	ns
Labile P (ppm)	0.07±0.01	0.07±0.01	ns	0.02±0.00	0.02±0.01	ns
Inorganic NaOH-extractable P (ppm)	1.37±0.59	1.27±0.42	ns	0.63±0.24	0.81±0.40	ns
Total NaOH-extractable P (ppm)	3.55±0.53	3.61±1.04	ns	1.04±0.24	1.16±0.20	ns
C/Total NaOH extractable P	0.47±0.17	0.52±0.07	ns	0.31±0.06	0.35±0.10	ns
Al (cmol_c_/kg soil)	0.97±0.17	0.80±0.10	ns	0.73±0.18	0.82±0.30	ns
Ca (cmol_c_/kg soil)	0.06±0.02	0.06±0.02	ns	0.01±0.01	0.02±0.00	ns
K (cmol_c_/kg soil)	0.10±0.02	0.10±0.02	ns	0.02±0.00	0.02±0.00	ns
Mg (cmol_c_/kg soil)	0.09±0.01	0.14±0.06	ns	0.02±0.01	0.02±0.00	ns
Na (cmol_c_/kg soil)	0.02±0.01	0.02±0.01	ns	0.01±0.01	0.02±0.00	ns
Ba (cmol_c_/kg soil)	<0.01	<0.01	ns	<0.01	<0.01	ns
Cu (cmol_c_/kg soil)	<0.01	<0.01	ns	<0.01	<0.01	ns
Fe (cmol_c_/kg soil)	0.23±0.04	0.29±0.03	ns	<0.01	<0.01	ns
Mn (cmol_c_/kg soil)	<0.01	<0.01	ns	<0.01	<0.01	ns
Ni (cmol_c_/kg soil)	<0.01	<0.01	ns	<0.01	<0.01	ns
Si (cmol_c_/kg soil)	0.05±0.02	0.06±0.01	ns	0.06±0.01	0.10±0.00	ns
Zn (cmol_c_/kg soil)	<0.01	<0.01	ns	<0.01	<0.01	ns

Top soil values were calculated by averaging values of the top depth classes: 0–5 cm, 5–10 cm, 10–20 cm and 20–30 cm. Deep soil were 150–200 cm. All values are expressed in mean ±95% confidence intervals (n = 3). Inorganic P and total P were extracted using NaOH. NS denotes there was non-significant difference between the two forest types based on the *t* test for matched paired comparison (n = 3). ns denotes the two forest types were insignificantly different.

The concentrations of Ca, Fe, K, Mg, Mn, Na and Ni decreased with depth for both forest types while those of Al, Ba, Na, Cu, Si and Zn remained relatively constant (Table S2). However, the monodominant forests had consistently lower concentrations of Ba, Mg and Ni than the mixed forests along the depth gradient (Table S2). None were significantly difference between the soils under the two forest types. Concentrations of other 7 elements from both forest types – B, Co, Cr, Mo, Se, Sr, Ti, and V – were at too low level to be detected by the CEC method.

However, prior to Bonferroni correction procedure, the top soils in the mixed forests had a significantly higher Ni concentration (P = 0.022) and their deep soils had a significantly higher level of Si (P = 0.030). These differences were likely the results of multiple tests of significance [30]. Moreover, these differences were unlikely to cause Ni and Si deficiencies in the plants within the monodominant forests (i.e., the differences were likely not biologically meaningful). In short, comparisons between the monodominant and mixed forests at top soils and deep soils show no significant or biologically meaningful differences in their soil properties.

Although we used a small number of replicate plots, our results are in accordance with other similar studies in the region. For example, Conway (1992) found that there were no differences in the mean values of the soil parameters – such as pH, organic C, N, total P, extractable P, K, Ca and Mg – between the monodominant forests and mixed forests at Ituri forest in the Democratic Republic of Congo, over 1000 km away from the Dja Faunal Reserve [10]. Similarly, in a different location at Ituri forest, Hart et al. (1989) showed that the soil factors – such as the concentrations of Ca, K, Mg and P in top soils (20-cm depth) and Ca, Mg and P in deep soils (150-cm depth) – were not different [34]. While acknowledging the paucity of studies, so far relatively few differences in soil properties were reported among the monodominant and mixed tropical forests [10]–[12]. Despite that, the findings of some soil parameters were not consistent across the studies in the same region. For example, in the same study as mentioned above, Hart et al (1989) found that the concentrations of K in deep soils between forest types were significantly different [34]. Also in another location at Ituri forest studied by Torti et al. (2001), the monodominant forests had a lower level (one-third) of nitrogen (ammonium and nitrate) availability in the soil than the mixed forests [9]. Such discrepancies suggest that we cannot assume all monodominant *G. dewevrei* forests to have the same soil properties as their adjacent mixed forests. Hence, it is important that future studies comparing the mixed and monodominant forest types investigate their soil characteristics.

This study shows that, generally, soils – including those at depth of 0–5 cm which are likely to be most influenced by the vegetation – were not different between the monodominant *G. dewevrei* forests and the adjacent mixed forests. Further, we have showed that, for the first time, the soil micronutrients were not different between these forest types. Thus, the edaphic conditions were unlikely to be the cause of monodominance in *G. dewevrei forests*. Nevertheless, there was variation among monodominant forest soils when comparing those from the Dja Faunal Reserve with the same *G. dewevrei* forest at Ituri [2]. The monodominant forests at Ituri had higher pH in the top (20-cm depth) soil (pH 4.17), greater proportion of sand (top soil  = 71.7%; 150 cm deep soil  = 68.4%), and higher concentrations of Ca (top soil  = 0.65 cmol_c_/kg; deep soil  = 0.76 cmol_c_/kg) and K (top soil  = 0.22 cmol_c_/kg; deep soil  = 0.12 cmol_c_/kg). This implies that the soil chemistry under the canopies of *G. dewevrei* forests is unlikely to be uniform.

Interestingly, these results also suggest that *G. dewevrei* did not influence soil chemistry in the monodominant forests. A handful of studies reported that the dominant vegetation could influence soil properties [16], [17] and these species-specific effects may be caused by inter-specific differences in uptake and storage of nutrients in above-ground biomass, input of nutrients from litter, litter characteristics, microbial association or organic acid exudation [15]. Nonetheless, this was not observed in our study. One possible reason is that other life forms might grow interspersed with and underneath the crowns of *G. dewevrei*, and the presence of these plants which vary in foliar nutrient contents may disrupt any effect of the dominant species on soil properties [15].

### Conclusions

We found no empirical evidence that the properties of the soil found under monodominant forests dominated by a single canopy tree species and adjacent forests not dominated by a single species in the Dja Faunal Reserve were significantly different. However, some differences in some soil parameters had been observed between both forest types at another central Africa site, Ituri, some 1000 km from our study site. Our results also highlight that *G. dewevrei* does not have strong influence on properties on surface soils. Torti *et al*. (2001) proposed that the turnover of nutrients in the monodominant forests is slowed down by the reduced leaf litter decomposition rate and thereby prevents the establishment of small-seeded species [9]. However, this mechanism proposed to explain monodominance is not consistent with our empirical observations which suggest that soils, or soil-vegetation interactions, are not the cause of difference in vegetation between the monodominant and mixed forest. Nevertheless, the discrepancy between this mechanism and our results does not necessarily mean that slow litter decomposition and slow nutrient turnover is unimportant when considering the mechanisms necessary for monodominance to arise [35]. Our results show that this soil-mediated mechanism alone is not sufficient to explain monodominance of *G. dewevrei* in Central African forests. Further research is required to understand the cause of classical monodominance, which likely means investigations into mechanisms that do not invoke major differences in soils to explain the visibly obvious differences in overlying vegetation. Other mechanisms proposed necessary for gaining recruitment advantages over other species to attain monodominance include a high canopy density that casts deep shade to out-compete light-demanding species; shade-tolerant saplings that enable survival and growth in the shade created by parent trees; ballistic dispersal that promotes gregarious habits for replacing individuals of other species; and ectomycorrhizal association which allows more efficient exploitation of larger volumes of soils or directly decompose leaf litter [9], [13].

## Supporting Information

Dataset S1 Original dataset for pH in water, bulk density, carbon (C) content, nitrogen (N) content, C/N ratio, labile phosphorus (P), inorganic NaOH-extractable P, total NaOH-extractable P, clay proportion, silt proportion, sand proportion, and particle size. G1, G2, G3 were forest plots dominated by *Gilbertiodendron dewevrei* and M1, M2, M3 were the adjacent higher-diversity forest plots where no species dominates. G1-M1, G2-M2, and G3-M3 were pairs of 1 ha plot.
(DOC)

Table S1 Detailed soil description of the pits (200-cm depth) from the three pairs of 1 ha forest plots at the Dja Faunal Reserve. The pairs were G1-M1, G2-M2, and G3-M3.
(DOC)

Table S2 Concentrations of Al, Ba, Ca, Cu, Fe, K, Mg, Mn, Na, Ni, Si and Zn found in soils at different depths sampled beneath plots of forest dominated by *Gilbertiodendron dewevrei* (G1, G2, G3) and adjacent higher-diversity forests where no species dominates (M1, M2, M3). G1-M1, G2-M2, and G3-M3 were pairs of 1 ha plot.
(DOC)
