# Supplementary material for: Soil Does Not Explain Monodominance in a Central African Tropical Forest
Source: PLoS One. 2011 Feb 10;6(2):e16996. doi: 10.1371/journal.pone.0016996 (PMC3037391; doi:10.1371/journal.pone.0016996)
Supplement: Dataset S1 — Original dataset for pH in water, bulk density, carbon (C) content, nitrogen (N) content, C/N ratio, labile phosphorus (P), inorganic NaOH-extractable P, total NaOH-extractable P, clay proportion, silt proportion, sand proportion, and particle size. G1, G2, G3 were forest plots dominated by Gilbertiodendron dewevrei and M1, M2, M3 were the adjacent higher-diversity forest plots where no species dominates. G1-M1, G2-M2, and G3-M3 were pairs of 1 ha plot. (DOC) [file pone.0016996.s001.doc]

|  |
| --- |
|  |
|  |
