# Supplementary material for: Soil Does Not Explain Monodominance in a Central African Tropical Forest
Source: PLoS One. 2011 Feb 10;6(2):e16996. doi: 10.1371/journal.pone.0016996 (PMC3037391; doi:10.1371/journal.pone.0016996)
Supplement: Table S1 — Detailed soil description of the pits (200-cm depth) from the three pairs of 1 ha forest plots at the Dja Faunal Reserve. The pairs were G1-M1, G2-M2, and G3-M3. (DOC) [file pone.0016996.s002.doc]

| Plot/Horizon | Description |
| --- | --- |
| G1 | Monodominant *G. dewevrei* forest. Topography was gentle undulating with some short terraces. Soil was very well drained, had low fertility and seemed to be a Ferralsol. A continuous pan of ironstones was present at 50 to 100 cm but roots and water could easily go through. Overall physical properties were good. |
| O horizon | Leaf litter over the soil with about 2.0 cm high, in different stages of decomposition ranging from leaves and twigs to humus. A large amount of fine roots was found inside and over the organic layer. |
| A_1_ [0–5 cm] | Organic matter content is high (4.9 % C). Soil was very well aggregated, showing stable, sub angular and granular structure, high plasticity, and high porosity, friable and soft. Bulk density was low (0.7 g cm^-3^). Texture was clay silt (25-52 % clay). Abundant faunal activity. Roots were very abundant in all size classes. Transition to the next layer is sharp and regular. ECEC 1.4 cmol_c_ kg^-1^, Al saturation 53 %. Colour was dusky red (10R 3/2). |
| A_2_ [5–12 cm] | Decreased in organic matter content (2.4 % C). Well aggregated, stable granular structure with some sub angular blocks, high plasticity, and high porosity, friable and soft but slightly firmer than A_1_. Bulk density was low (0.8 g cm^-3^). Texture was clay silt (25-52 % clay). Less roots but still abundant. Transition to the next layer was sharp and regular. ECEC 1.3 cmol_c_ kg^-1^, Al saturation 80 %. Colour was dark reddish brown (2.5R 3/4). |
| B_1_ [12–45 cm] | Low organic matter content (1 % C). Well aggregated, predominantly stable granular structure, high plasticity, and high porosity. Well drained, friable and soft. Bulk density was low (1.0 g cm^-3^). Texture was clay silt (25-52 % clay). Abundant faunal activity. Roots were abundant. Typical ferralic horizon. Homogeneous, transition to the next layer was diffuse and wavy (varying from 32 to 45 cm deep). ECEC 0.9 cmol_c_ kg^-1^, Al saturation 87 %. Colour was red (10R 4/6). |
| B_2_ [45–82 cm] | Scattered small petroplinthite (ironstones). Despite the presence of petroplinthite, soil was well aggregated, stable granular structure, has high plasticity, and very high porosity. Well drained, friable and soft. Bulk density was very low. Texture was clay silt (25-52 % clay). Abundant faunal activity with termite nest. Roots were still abundant. Typical ferralic horizon. Homogeneous and uniform, distinction between soil layers was difficult; transition among layers was diffuse and very irregular (wavy, varying from 39 to 82 cm deep). Colour was red (10R 4/6). |
| B_3_ [82–127 cm] | Continuous petroplinthite hardpan (ironstones). Ironstone size increased with depth. Roots could go through the hardpan, and fine roots predominated. Well drained. Bulk density was very low (0.8 g cm^-3^). Texture was clay silt (25-52 % clay). Typical ferralic horizon. Homogeneous and uniform, distinction between soil layers was mostly due to presence of petroplinthite. Transition among layers is wavy (varying from 63 to 127 cm deep). Colour was red (10R 4/6). |
| B_4_ [>127 cm] | Low organic matter content (0.5% C). Continuous petroplinthite hardpan (ironstones), but stones had larger size and shorter space between stone clusters. Roots could still go through the hardpan but they were few and fine. Well drained, bulk density was low. Texture was clay silt (25-52 % clay). Typical ferralic horizon. Homogeneous and uniform. ECEC 0.7 cmol_c_ kg^-1^, Al saturation 89 %. Colour was red (10R 4/6). |
|  |  |
| M1 | Mixed species forest, adjacent to the monodominant plot G1 (452 m apart). Topography was flat. Soil was very similar to that found in G1. It was well drained, had low fertility and seemed to be a Ferralsol. A continuous pan of ironstones was also present but usually slightly deeper (about 100 cm). Roots and water could easily go through. Overall physical properties were good. |
| O horizon | Leaf litter over the soil with about 2.0 cm high, in different stages of decomposition ranging from leaves and twigs to humus. A large amount of fine roots was found inside and over the organic layer. |
| A_1_ [0–3 cm] | Organic matter content was high (4.0 % C). Soil was very well aggregated, with stable, sub angular and granular structure, high plasticity, and very high porosity, friable and soft. Bulk density was low (0.7 g cm^-3^). Texture was clay silt (25-52 % clay). Abundant faunal activity. Roots were very abundant in all size classes. Transition to the next layer was sharp and regular. ECEC 1.2 cmol_c_ kg^-1^, Al saturation 54%. Colour was dusky red (10R 3/4). |
| A_2_ [3–12 cm] | Decreased in organic matter content (2.2 % C). Well aggregated, stable granular structure, high plasticity, and high porosity, friable and soft but slightly firmer than A_1_. Bulk density was low (0.8 g cm^-3^). Texture was clay silt (25-52 % clay). Less roots but still abundant. Transition to the next layer was diffuse and irregular (wavy). ECEC 1.1 cmol_c_ kg^-1^, Al saturation 81%. Colour was red (10R 4/6). |
| AB [12–18 cm] | Relatively high organic matter content (1.5 % C). Well aggregated, predominantly stable granular structure, high plasticity, and high porosity. Well drained, friable and soft. Bulk density was low (0.9 g cm^-3^). Texture was clay silt (25-52 % clay). Roots were abundant but comparatively less than in G5. Typical ferralic horizon. Homogeneous, transition to the next layer was diffuse and regular. ECEC 1.0 cmol_c_ kg^-1^, Al saturation 81%. Colour was red (10R 4/6). |
| BA [18–25 cm] | Lower organic matter content (1 % C). Well aggregated, predominantly stable granular structure, high plasticity, and high porosity. Well drained, friable and soft. Bulk density was low. Texture is clay silt (25-52 % clay). Roots were many. Typical ferralic horizon. Homogeneous, transition to the next layer was diffuse and regular. ECEC 1.1 cmol_c_ kg^-1^, Al saturation 91%. Colour was red (10R 4/6). |
| B_1_ [25–73 cm] | Well aggregated, stable granular structure, high plasticity, and very high porosity. Well drained, friable and soft. Bulk density was very low. Texture was clay silt (25-52 % clay). Still some roots. Typical ferralic horizon. Soil was very homogeneous and uniform; no changes in structure with depth. Transition among layers was diffuse and irregular (wavy). Colour was red (10R 4/6). |
| B_2_ [73–300 cm] | Soil was still very well aggregated, with stable granular structures, high plasticity, and very high porosity. Well drained, friable and soft. Bulk density was low (1.0 g cm^-3^). Texture was clay silt (25-52 % clay). Roots were present in the entire profile but were few below 1 m. Typical ferralic horizon. Soil was very homogeneous and uniform; no changes in structure with depth. Transition among layers was diffuse and irregular (wavy). ECEC 0.7 cmol_c_ kg^-1^, Al saturation 88%. Colour was red (10R 4/6). |
| B_3_ [>300 cm] | Continuous petroplinthite hardpan (ironstones). Fine roots were found through the hardpan. |
| G2 | Monodominant *Gilbertodendron dewevrei* forest. Topography was gentle undulating. Most of the area was well drained but approximately 1/5 of the plot showed hydromorphic features at depths greater than 1.5 m. A continuous pan of ironstones appeared in the subsoil, with depth varying from 30 to >200 cm, but this did not seem to constrain root growth or drainage. Where hydromorphic features were present, it seemed to be related to very deep impermeable layers (>300 cm). The pit information did not represent well for this plot; most of the area was similar to the soils described for the plot G1. Overall physical properties were good; soil was soft and friable. It had low fertility and seemed to be a Ferralsol, even in the lower drainage areas. |
| O horizon | Leaf litter over the soil was approximately 5.0 cm thick in different stages of decay ranging from intact leaves and twigs to humus. A large amount of fine roots was found inside and over the organic layer. |
| A_1_ [0–6 cm] | Organic matter content was high (3.8 % C). Soil was very well aggregated, showing a stable, sub-angular and granular structure. It had high porosity, and was friable and soft. Bulk density was distinctively low (0.4 g cm^3^). Texture was clay (25-52 % clay). Roots were very abundant. Transition to the next layer was sharp and regular. ECEC 1.6 cmol_c_ kg^-1^, Al saturation 69 %. Colour was dark brown (10YR 3/3). |
| A_2_ [6–12 cm] | Decrease in organic matter content (2.4 % C). Well aggregated, stable granular structure; high porosity, friable and soft but slightly firmer than A_1_. Bulk density was low (0.7 g cm^-3^). Texture was clay silt (25-52 % clay). Roots were still abundant. Transition to the next layer was sharp and regular. ECEC 1.4 cmol_c_ kg^-1^, Al saturation 84 %. Colour was yellowish brown (10YR 5/4). |
| AB [12–18 cm] | Decrease in organic matter content (1 % C). Well aggregated, predominantly stable granular structure; and high porosity. Well drained, friable and soft. Bulk density was low (1.1 g cm^-3^). Texture was clay silt (25-52% clay). Some roots. Transition to the next layer was diffuse and regular. ECEC 0.9 cmol_c_ kg^-1^, Al saturation 87%. Colour was brownish yellow (10YR 5/6). |
| B_1_ [18–82 cm] | Reduction in aggregation, but still some granular structure; plastic; drainage was good but become moderate below this layer. Bulk density was moderate to high (1.3 g cm^-3^). Texture is clay (25-52 % clay). Some roots. Still a ferralic horizon. Homogeneous, transition to the next layer was diffuse and regular. Colour was brownish yellow (10YR 6/6). |
| B_2_ [82–168 cm] | Greater reduction in aggregation, and greater loss in structure; plastic; drainage was moderate. Some mottling was present but its extent was moderate. Small increase in bulk density; Texture was clay (25-52 % clay); Few roots; some transition between ferralic horizon and gleyic colour pattern. Homogeneous, transition to the next layer was diffuse and regular. Colour was yellow (10YR 8/6). |
| C_1_ [>168 cm] | Greater reduction in aggregation and greater loss in structure; plastic; drainage was poor. Hydromorphic features dominated with gleyic colour patterns. Plinthite was scattered through the horizon; large increase in bulk density (1.4 g cm^-3^); Texture was silty clay (25-52 % clay); roots were rare; colour was grey with mottling (10YR 6/1). |
|  |  |
| M2 | Mixed forest, adjacent to the monodominant plot G2 (505 m apart). Topography was gentle undulating. Soil was well drained, but there were some places with deep hydromorphic features, similar to G2; had low fertility and seemed to be a Ferralsol. A continuous pan of ironstones was present, roots and water could go through. However there was likely root restriction to the top soil. |
| O horizon | Leaf litter over the soil with about 2.0 cm high, in different stages of decomposition ranging from leaves and twigs to humus. A large amount of fine roots was found inside and over the organic layer. |
| A_1_ [0–5 cm] | Organic matter content was moderate high (2.7 % C). Soil was well aggregated, with sub angular and granular structures but these were not very stable; moderate plasticity; high porosity; friable and soft. Bulk density was low (0.6 g cm^3^). Texture was sandy clay (25-52 % clay). Roots were very abundant. Transition to the next layer was sharp and regular. ECEC 1.2 cmol_c_ kg^-1^, Al saturation 52 %. Colour was yellowish red (5YR 4/6). |
| A_2_ [5–10 cm] | Decrease in organic matter content (1.6% C). Reduction in structure, with dominance of granular aggregates; moderate plasticity; small decrease in porosity; still friable and soft but slightly less than A_1_. Consequent increase in bulk density (1.1 g cm^-3^). Texture was clay silt (25-52% clay). Less roots but still abundant. Transition to the next layer was diffuse and slightly irregular (wavy). ECEC 1.1 cmol_c_ kg^-1^, Al saturation 78%. Colour was yellowish red (5YR 5/6). |
| AB [10–22 cm] | Small decrease in organic matter content (1.3 % C). No significant change in structure; dominance of granular aggregates; moderate plasticity; small decrease in porosity; less friable than upper layers. Small increase in bulk density (1.2 g cm^-3^). Texture was clay silt (25-52 % clay). Less roots. Transition to the next layer was diffuse and slightly irregular (wavy). ECEC 0.8 cmol_c_ kg^-1^, Al saturation 81 %. Colour was yellowish red (5YR 5/6). |
| B_1_ [22–51 cm] | Small decrease in organic matter content (1.0 % C). Granular aggregation; moderate plasticity. No significant change in soil structure. Well drained. No change in bulk density (1.2 g cm^-3^). Texture was clay silt (25-52 % clay). Some roots. Typical ferralic horizon. Soil was very homogeneous and uniform; Transition to next layer was abrupt and slightly irregular (wavy). ECEC 0.7 cmol_c_ kg^-1^, Al saturation 82 %. Colour was yellowish red (5YR 5/8). |
| B_2_ [51–110 cm] | Petroplinthic hardpan, with small rounded shape, not cemented. Roots still get through but most were restricted to upper layers. Well drained. Texture was clay silt (25-52 % clay). Few roots, apparent constrained by hardpan. Typical ferralic horizon. Soil was very homogeneous and uniform; transition among layers was diffuse and regular. Colour was red (7.5R 4/6). |
| B_3_ [>110 cm] | Continuous petroplinthite hardpan (petroplinthic layer), taking about 80 % of soil volume. Organic matter content was low (0.4 % C). Drainage was good. Roots were rare but still present. ECEC 0.8 cmol_c_ kg^-1^, Al saturation 91 %. Dark red (7.5R 3/4). |
|  |  |
| G3 | Monodominant *G. dewevrei* forest. Topography was flat. Most of the area was moderate to poorly drained with hydromorphic features at depths greater than 1 m. Water table was found at 180 cm during dry season, and might rise to about 1 m. There was however still good soil structure in the top 1 m. Overall physical properties of top 1 m were good. Soil was soft and friable; had moderate drainage and low fertility and seemed to be a gleysol. At this well drained plot, petroplinthite was often found at about 1m, here, such petroplinthic layer was found as plinthite, this might indicate that our study sites were once located at lower ground level, with fluctuating water tables – a condition necessary to the segregation of iron and formation of plinthite. With time, the erosion process might have placed these areas in upper land, changing their drainage and moisture regime, and thus favouring the conversion of plinthite to petroplinthite. |
| O horizon | Leaf litter over the soil with about 3.0 cm high, in different stages of decomposition ranging from leaves and twigs to humus. A large amount of fine roots was found over the organic layer forming a root mat. |
| A_1_ [0–7 cm] | Organic matter content was very high (7.2 % C). Soil was very well aggregated, showing a stable, block structure. It had high porosity, was friable and soft. Bulk density was distinctively low (0.4 g cm^-3^). Texture was clay (25-52 % clay). Roots were distinctively abundant. Transition to the next layer was abrupt and regular. ECEC 1.3 cmol_c_ kg^-1^. Al saturation 49 %. Colour was very dark greyish brown (10YR 3/2). |
| A_2_ [7–14 cm] | Large decrease in organic matter content (2.6 % C). Soil structure changes to dominance of granular aggregates; lower porosity than A_1_ but still friable and soft. Bulk density was low (0.8 g cm^-3^). Clay increased with depth. Roots were still abundant. Transition to the next layer was sharp and regular. ECEC 1.4 cmol_c_ kg^-1^, Al saturation 78 %. Colour was dark greyish brown (10YR 4/2). |
| B_1_ [14–47 cm] | Organic matter content was low (about 1% C). Reduction in aggregation, but still some block structure; not very plastic; drainage was good to moderate. Bulk density was moderate to high (1.3 g cm^-3^). Clay increased with depth. Texture was sandy clay (25-52% clay). Homogeneous; transition to the next layer was sharp and regular. Colour was brown (10YR 5/3). |
| B_2_ [47–78 cm] | Greater reduction in aggregation, and greater loss in structure; drainage was moderate. Small increase in bulk density; Clay increased with depth; Roots were rare; some transition between argic horizon and gleyic colour pattern. Homogeneous; transition to the next layer was diffuse and regular. Colour was yellowish brown (10YR 5/4). |
| BC [78–144 cm] | Greater reduction in aggregation and greater loss in structure; plastic; drainage was poor. Hydromorphic features dominated with gleyic colour patterns. Plinthite was scattered through the horizon; large increase in bulk density; Texture was silty clay (25-52 % clay); roots were rare; colour was very pale brown with mottling (10YR 8/4). |
| C_1_ [>144 cm] | Soil organic matter was very low (0.2 % C). Greater reduction in aggregation and greater loss in structure; very plastic; drainage was very poor. Hydromorphic features dominate with gleyic colour patterns. Plinthite was scattered through the horizon; large increase in bulk density (1.5 g cm^3^); Texture was silty (25-52 % clay); colour was grey with mottling. |
|  |  |
| M3 | Mixed forest, adjacent to the monodominant plot G3 (818 m apart). Topography was flat. Soil very similar to G3, it was moderate to poorly drained, with deep hydromorphic features. Water table was found at 180 cm during dry season, and might rise to about 1 m. There was however still good soil structure in the top 1 m, and physical properties of top 1 m were good. Soil was soft and friable and had moderate drainage. It had low fertility and seemed to be a gleysol. At the well drained plots, petroplinthite was often found at about 1m, here and in G3, such petroplinthic layer was found as plinthite. |
| O horizon | Leaf litter over the soil with about 2.0 cm high, in different stages of decomposition ranging from leaves and twigs to humus. A large amount of fine roots was found over the organic layer. |
| A_1_ [0–4 cm] | Organic matter content was moderate high (4.3 % C). Soil aggregation was moderate, with block structures but these were not very stable; moderate plasticity; still friable and soft. Bulk density was low (0.7 g cm^-3^). Texture was sandy clay (25-52 % clay). Roots were very abundant. Transition to the next layer was sharp and regular. ECEC 1.5 cmol_c_ kg^-1^, Al saturation 37 %. Colour was very dark greyish brown (10YR 3/2). |
| A_2_ [4–9 cm] | Significant decrease in organic matter content (1.7 % C). Reduction in structure, with little aggregation; moderate plasticity; decrease in porosity; Consequent increase in bulk density (1.3 g cm^-3^). Texture was sandy clay (25-52 % clay). Less roots but still abundant. Transition to the next layer was diffuse and regular. ECEC 1.2 cmol_c_ kg^-1^, Al saturation 80 %. Colour was dark greyish brown (10YR 4/2). |
| AB [9–16 cm] | Small decrease in organic matter content (1.3 % C). No significant change in structure; dominance of granular aggregates; moderate plasticity; small decrease in porosity; less friable than upper layers. Small increase in bulk density (1.3 g cm^3^). Texture was clay sand (25-52 % clay). Less roots. Transition to the next layer was diffuse and regular. ECEC 1.2 cmol_c_ kg^-1^, Al saturation 80 %. Colour was brown (10YR 5/3). |
| B_1_ [16–68 cm] | Decrease in organic matter content (0.7 % C). Reduction in aggregation; loss of structure; moderate plasticity. Moderately drained. Increment in bulk density (1.5 g cm^-3^). Texture was clay sand (25-52 % clay). Few roots. Soil was very homogeneous and uniform; Transition to next layer was diffuse and regular. ECEC 1.1 cmol_c_ kg^-1^, Al saturation 89 %. Colour was red (10R 4/6). |
| B_2_ [68–131 cm] | No significant reduction in aggregation or structure; moderate plasticity. Moderately drained. Texture was clay sand (25-52 % clay). Few roots. Soil was very homogeneous and uniform; Transition to next layer is diffuse and regular. |
| BC [131–153 cm] | Greater reduction in aggregation and greater loss in structure; plastic; drainage was poor. Hydromorphic features dominated with gleyic colour patterns. Plinthite was scattered through the horizon; large increase in bulk density; Texture was silty clay (25-52 % clay); roots were rare; colour was yellowish brown with mottling (10YR 5/4). |
| C_1_ [>153 cm] | Soil organic matter content was very low (0.2 % C). Greater reduction in aggregation and greater loss in structure; very plastic; drainage was very poor. Hydromorphic features dominated with gleyic colour patterns. Plinthite was scattered through the horizon; slightly decreased in bulk density (1.3 g cm^-3^); Texture was silty (25-52 % clay); ECEC 1.1 cmol_c_ kg^-1^, Al saturation 93 %; colour was grey with mottling. |
