# Supplementary material for: Soil Does Not Explain Monodominance in a Central African Tropical Forest
Source: PLoS One. 2011 Feb 10;6(2):e16996. doi: 10.1371/journal.pone.0016996 (PMC3037391; doi:10.1371/journal.pone.0016996)
Supplement: Table S2 — Concentrations of Al, Ba, Ca, Cu, Fe, K, Mg, Mn, Na, Ni, Si and Zn found in soils at different depths sampled beneath plots of forest dominated by Gilbertiodendron dewevrei (G1, G2, G3) and adjacent higher-diversity forests where no species dominates (M1, M2, M3). G1-M1, G2-M2, and G3-M3 were pairs of 1 ha plot. (DOC) [file pone.0016996.s003.doc]

|  |
| --- |

|  |
| --- |

|  |
| --- |
